# Loneliness and Patterns of Health Service Usage: Evidence From Healthy Ageing in Scotland

**DOI:** 10.1093/geroni/igaa057.2206

**Published:** 2020-12-16

**Authors:** Elaine Douglas, David Bell

**Affiliations:** University of Stirling, Stirling, Scotland, United Kingdom

## Abstract

Loneliness is associated with poorer health status and health outcomes. Yet, little is known about how loneliness in ageing populations is associated with health service usage. Loneliness (UCLA-3) was measured in older people in Scotland (Healthy Ageing in Scotland, HAGIS, n = 1,057). We analysed socio-demographic, perceived health, and health behaviour characteristics using descriptive statistics and logistic regression. The survey data (HAGIS, 2016/17) were linked to retrospective administrative health data to investigate patterns of health service usage (from 2005), such as the number of hospital visits and mean length of stay, and their associated costs. Two-part models were used to highlight variation i) in those who had ever vs never been admitted to hospital, and ii) between those who had been admitted. Our results highlight the variation in hospital service usage in those experiencing loneliness and opens discussion on the implications for older people and hospital services.

